# Effects of periodontitis on postoperative pneumonia in patients with lung and esophageal cancer

**DOI:** 10.1111/1759-7714.13828

**Published:** 2021-01-24

**Authors:** Chunling Jia, Yijun Luan, Xibo Li, Xiaoying Zhang, Cuirong Li

**Affiliations:** ^1^ Department of Oral Medicine Qilu Hospital of Shandong University Jinan China; ^2^ Institute of Stomatology Shandong University Jinan China; ^3^ Department of Thoracic Surgery Qilu Hospital of Shandong University Jinan China; ^4^ Department of Geriatrics Qilu Hospital of Shandong University Jinan China; ^5^ Department of Stomatology Qilu Hospital of Shandong University Jinan China

**Keywords:** Chronic periodontitis, esophageal cancer, lung cancer, pathogenic bacteria, periodontal pocket

## Abstract

**Background:**

Few studies have been conducted on the relationship between chronic periodontitis and postoperative pneumonia (POP) in patients with lung and esophageal cancer. Furthermore, it remains controversial as to whether improving the periodontal condition of patients with lung and esophageal cancer before surgery reduces the incidence of POP. This retrospective study was conducted to assess the effects of periodontal therapy in patients with lung and esophageal cancer to prevent POP.

**Methods:**

A total of 265 patients with lung or esophageal cancer complicated with chronic periodontitis who underwent open thoracotomy between July 2015 and June 2019 were selected and given the choice of being in the experimental or control group. A total of 141 participants in the experimental group received periodontal therapy, and 124 participants in the control group did not receive periodontal therapy. All clinical data of participants in both groups were retrospectively studied to determine the incidence of POP on the 30th day after discharge from hospital.

**Results:**

Eight patients in the experimental and six in the control group, respectively, were excluded from the study. It was found that four of the 133 patients suffered from POP in the experimental group (incidence: 3.01%). A total of 18 of 118 patients in the control group had a pulmonary infection (incidence: 15.25%). POP incidence in the experimental group was significantly lower than that in the control group, and in the level analysis of different types of periodontitis, surgical methods, and diseases (*p* < 0.05).

**Conclusions:**

Periodontal treatment is associated with a lower incidence of POP following lung and esophageal cancer surgery. Improving the periodontal condition of patients helps prevent POP. The presence of periodontitis is an important predisposing factor for POP in patients after open thoracotomy. Periodontal examination and therapy are recommended before the surgical treatment of lung or esophageal cancer.

## Graphical abstract

Periodontal treatment is associated with a lower incidence of POP following lung and esophageal cancer surgery, and it is recommended that periodontal examination and therapy be carried out before surgical treatment of lung or esophageal cancer.

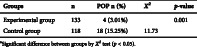

## INTRODUCTION

The oral cavity directly connects with the respiratory tract. Oral disease, especially periodontal disease, which has a high morbidity rate in humans, is related to the whole body. As a chronic oral inflammatory disease, periodontitis is a common plaque‐induced periodontal infection that is a major cause of tooth loss worldwide. Inflammation of the periodontium, formation of the periodontal pocket, progressive loss of connective tissue attachment, and resorption of alveolar bone are the main clinical manifestations of chronic periodontitis. It is initiated and sustained by dental plaque biofilms. Being tooth‐borne, complex microbial communities, dental plaque, especially the subgingival plaque in periodontal pockets, are colonized by a large variety of flora related to pneumonia, such as *A. actinomycetemcomitans*, *Actinomyces israelii*, *Capnocytophaga* spp., *Eikenella corrodens*, *Prevotella intermedia*, and *Streptococcus constellatus*.^1^ These periodontal bacteria have strong associations with pneumonia and other systemic inflammatory conditions.^2^, ^3^, ^4^, ^5^ They can easily penetrate the microvessels of periodontal pockets to cause bacteremia when patients undergo dental treatment or with tooth brushing or mastication.^6^ In addition, bacteria in dental plaque can be shed into the saliva and then aspirated into the lower respiratory tract and lung to cause infection. Thus, bacteria from the periodontally diseased pockets are an important resource for respiratory infections. Periodontal pockets may serve as a persistent reservoir of potential pathogens for respiratory infections. Patients undergoing surgery are at risk of developing postoperative respiratory infections from periodontitis.^7^

Postoperative pneumonia (POP) is an important infectious complication in patients with lung or esophageal cancer following open thoracotomy and the main cause of death in the perioperative period.^8^ Studies have reported that bacterial pathogens linked to POP are composed of endogenous, hospital‐related, and drug‐resistant microbes.^9^ It has also been reported that patients with poor oral hygiene and periodontal condition have significantly higher rates of pneumonia.^10^, ^11^ Pulmonary infection rates in the elderly and those in intensive care units have reduced significantly after treatment to improve adverse oral environments.^12^ Patients who had undergone dental plaque control by a dentist were reported to have a lower incidence of POP.^13^ Many studies have shown that the oral cavity may be the main endogenous pathogen reservoir of POP.^14^ Periodontal disease is a risk factor for infectious complications after surgery.^15^ There has been a high incidence of periodontitis reported among patients with lung or esophageal cancer.^16^ However, the effects of treating chronic periodontitis in patients with lung or esophageal cancer on POP has been rarely studied.^13^, ^17^ As few clinical guidelines for preventing POP recommend oral examination or treatment as routine preoperative care, it remains controversial whether improving patients' periodontal condition assists in lowering the incidence of POP before surgery.^18^

The treatment of chronic periodontitis aims to control the bacterial challenge characteristic of periodontitis while addressing local risk factors. It is directed against the disruption, removal, and control of the subgingival plaque biofilm located in the periodontal pocket. Alteration or elimination of periodontal pathogens and resolution of inflammation are the key objectives to create an environment conducive to periodontal health.^19^ This study retrospectively evaluates the effects of periodontal treatment on reducing POP incidence in patients following lung or esophageal cancer surgery.

## METHODS

Each patient signed a consent form prior to entering the study. The study complied with the Helsinki Declaration of 1975 as revised in 2000. The study protocol was reviewed and approved by the Ethics Committee of Qilu Hospital of Shandong University.

All patients with lung or esophageal cancer who underwent thoracotomy at the Thoracic Surgery Department of Qilu Hospital of Shandong University between July 2014 and June 2019 were selected.

The inclusion criteria were as follows: all participants were confirmed on imaging and pathological biopsy to have primary lung or esophageal cancer without distant metastasis and were eligible for an open thoracotomy. Patients who had other conditions such as diabetes mellitus (DM), hypertension, chronic obstructive pulmonary disease (COPD) that may influence the efficacy of the operation were excluded from the study. Preoperative pulmonary function, each index of laboratory testing, and electrocardiogram (EKG) results were normal. None of the participants received neoadjuvant chemotherapy or previous radiotherapy, had >15 natural teeth with each quadrant having ≥3, were between 18 and 70 years, with no smoking during the study period, and had chronic periodontitis.

Criteria for POP: Patients with three or more of the following indicators were considered to have POP.^20^, ^21^ (i) Patients had a fever (T > 38°C) 72 h after surgery or once more within 72 h; (ii) increased white blood cell count (>12 × 10^9^/L–15 × 10^9^/L), or second increase (>10 × 10^9^/L) after it returned to normal; (iii) chest x‐ray showed consolidation or increasing patchy shadows of the lung tissue; and (iv) patients coughed up purulent sputum or had a positive sputum culture. Patients who met four criteria and one other criterion were considered to have POP.

Oral examinations were conducted by an experienced dentist and the assessments consisted of the presence and degree of periodontitis. Periodontal clinical measurements were performed at six sites per tooth and included the probing depth (PD), clinical attachment level (CAL), bleeding on probing (BOP), and suppuration (SUP).

Criteria for periodontitis: the severity of periodontitis was categorized as mild, moderate, or severe as follows: mild, gingival tissue was inflamed, BOP (+), PD < = 4 mm, 1 or 2 mm of CAL; moderate, gingival tissue was inflamed, BOP (+), or SUP, PD < = 6 mm, 3 or 4 mm of CAL; and

severe, gingival tissue was inflamed, BOP (+), or SUP, PD >6 mm, 5 mm or more of CAL.

Only patients with plaque and calculus visibly present and periodontal disease affecting more than 30% of the sites were enrolled.

A total of 265 patients with chronic periodontitis were divided into two groups according to their preference: 141 participants received periodontal treatment (experimental group), and 124 participants did not receive periodontal treatment (control group). There was no significant difference with regard to age, sex distribution, periodontal status, and other factors between the two groups (Tables 1 and 2).

**TABLE 1:** Characteristics of all participants in the study

Parameters	Experimental group (*n* = 133)	Control group (*n* = 118)
Age (years; mean ± SD)	56.4 ± 13.3	55.1 ± 10.9
Males (n)	82	73
Females (n)	51	45
**Chronic periodontitis**		
Mild (n)	32	28
Moderate (n)	56	48
Severe (n)	45	42
**Lung cancer (total)**	78	67
Wedge resection (n)	15	12
Segmentectomy (n)	22	20
Sleeve resection (n)	18	18
Lobectomy (n)	23	17
**Esophageal cancer (total)**	55	51
Cervical anastomosis (n)	13	11
Above aortic arch (n)	15	16
Below aortic arch (n)	27	24
**Initial PFT (mean ± SD)**		
FVC (% pred)	101.4 ± 16.2	102.0 ± 15.8
FEV1 (% pred)	102.6 ± 20.8	102.7 ± 18.5
FEV1/FVC > 80%, n (%)	98 (100%)	87 (100%)
DL co (% pred)	97.2.0 ± 17.1	96.4 ± 16.7
DL co > 80% pred, n (%)	98 (100%)	87 (100%)
**Smoking (n)**	47	39
**Charlson comorbidity score, n (%)**		
Score 0–1	75 (56.4%)	65(55.1%)
Score 2	58 (43.6%)	53 (44.9%)
Score > 2	0	0
**Rarely visited dentist, n (%)**	124 (93.2%)	109 (92.4%)
**Drain tube (days) mean ± SD**	4.9 ± 2.1	5.0 ± 2. 0
**Days in hospital (mean ± SD)**	20.9 ± 7.8	21.5 ± 6.3

Note: Every parameter with mean ± SD had no significant difference between the groups by *t*‐test (*p >* 0.05). Every parameter with n (%) had no significant difference between the groups by *X* ^2^ test (*p >* 0.05).

**TABLE 2:** Periodontal index of all study participants

Parameter	Experimental group (before treatment)	Experimental group (after treatment)	Control group
PD (mm; mean ± SD)	5.2 ± 0.7^a^	2.2 ± 0.7^b^	5.3 ± 0.6
CAL (mm; mean ± SD)	4.7 ± 0.5^a^	3.3 ± 0.5^b^	4.6 ± 0.5
BOP (% site; mean ± SD)	2.5 ± 4.8^a^	0^b^	2.3 ± 4.9
SUP (% site; mean ± SD)	2.0 ± 2.9^a^	0^b^	2.1 ± 3.4

a Every parameter had no significant difference between the experimental group (before treatment) and control group by *t*‐test (*p >* 0.05).

b Every parameter had significant difference between the experimental group (after treatment) and control or experimental group (before treatment) by *t*‐test (*p <* 0.05).

Abbreviations: BOP, bleeding on probing; CAL, clinical attachment level; PD, probing depth; SUP, suppuration.

Therapy for periodontitis: participants in the experimental group received nonsurgical therapy from a dentist seven days before surgery. The same dentist was responsible for giving participants an oral examination, oral health education tailored to their specific situation, removal of some teeth with no reserve value, filling of cavities, and temporary fixation of loose teeth. Full‐mouth scaling, root planing, polishing, and medicine placement in the periodontal pockets were performed. The periodontal pockets were flushed alternately with a 3% hydrogen peroxide solution and 0.9% sodium chloride. A periodontal gel (antibacterial peptide, made by Beijing Ruise Biological Technology Co.) was then carefully injected into the bottom of the periodontal pocket until it overflowed from the opening of the pocket, and a cotton ball was gently used to press the gum to the original position. The above medical procedure was repeated once every two days until the patient had no gingival bleeding, suppuration, and dental plaque before surgery. During the perioperative period, patients were asked to brush their teeth using the Bass method three times a day. Toothbrushing was performed half an hour after each meal and lasted >3 min so that every tooth would be brushed effectively. A red neural solution was used to disclose the dental plaque. Dental floss and toothpicks were used for interdental surface cleaning. A mouth rinse (10 ml normal saline) was used three times a day between every two meals.

Oral visits were performed on the seventh day after surgery and the day before discharge from the hospital. Participants in the control group did not receive any special oral care and carried out their routine daily oral procedures. The periodontal index of all participants in the study is shown in Table 2. There was a significant statistical difference between the periodontal index before and after periodontal treatment in the experimental group (*p* < 0.05).

The clinical data of all participants in both groups were collected retrospectively on the 30th day of discharge from hospital (Tables 1 and 2).

**Statistical analysis**

All data were analyzed using Stata version 15.1. Continuous variables were expressed as the mean ± standard deviation. A Student's *t*‐test was used to analyze continuous variables. A *X* ^*2*^ test or stratified analysis was used to compare categorical variables between groups. A *p*‐value of <0.05 was considered statistically significant.

## RESULTS

Among the 141 participants in the experimental group, three were excluded as they were found to have gingival bleeding or dental plaque at the oral visits after the surgery. Five participants in the experimental group and six in the control group were excluded for the following reasons: surgery time > 4 h,^20^ inability to remove the drainage tube on the seventh day after surgery, and complications such as bronchial stump fistula and anastomotic fistula. All participants were evaluated on the 30th postoperative day.

Four of the 133 patients suffered from POP in the experimental group (incidence: 3.01%). A total of 18 of 118 patients in the control group had a pulmonary infection (incidence: 15.25%). POP incidence in the experimental group was significantly lower than that in the control group, and this difference was statistically significant (*p* < 0.05, Table 3).

**TABLE 3:** Incidence of postoperative pneumonia (POP) of all study participants

Groups	n	POP n (%)	*X* ^*2*^	*p*‐value
Experimental group	133	4 (3.01%)		0.001^a^
Control group	118	18 (15.25%)	11.73	

^a^ Significant difference between groups by *X* ^*2*^ test (*p <* 0.05).

**FIGURE 1:**
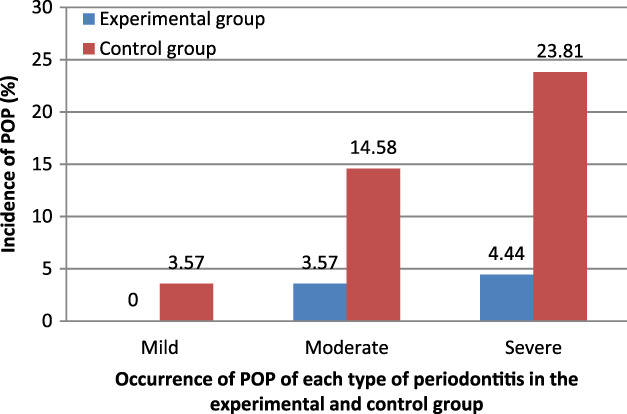
Occurrence of postoperative pneumonia (POP) of each type of periodontitis in the experimental and control groups. *Significant difference between two groups by *X* ^2^ test of Mantel–Haenszel–Cochran (*p* < 0.05)

In the experimental group, no patients had mild POP, but 2 patients had moderate POP, and there were 2 patients with severe periodontitis. In the control group there was one patient with mild POP, seven with moderate POP and 10 with severe periodontitis in the control group, respectively. POP incidence in the experimental group was significantly different from that in the control group (*p* < 0.05; Figure 1 and Table 4).

**TABLE 4:** Postoperative pneumonia (POP) in all study participants

Parameters	Experimental group	Control group	*X* ^*2*^	*p*‐value
Periodontitis				
Mild	0	1	11.69	0.0006^a^
Moderate	2	7		
Severe	2	10		
Lung cancer	3	12	11.83	0.0006^a^
Sophageal cancer	1	6		
Lung surgery				
Wedge resection	0	1		
Lobectomy	1	2	7.16	0.0075^a^
Segmentectomy	1	4		
Sleeve resection	1	5		
Esophageal surgery				
Cervical anastomosis	0	1	4.14	0.0419^a^
Above aortic arch	0	2		
Below aortic arch	1	3		

^a^ Significant difference between two groups by *X* ^2^ test of Mantel–Haenszel–Cochran (*p* < 0.05).

**FIGURE 2:**
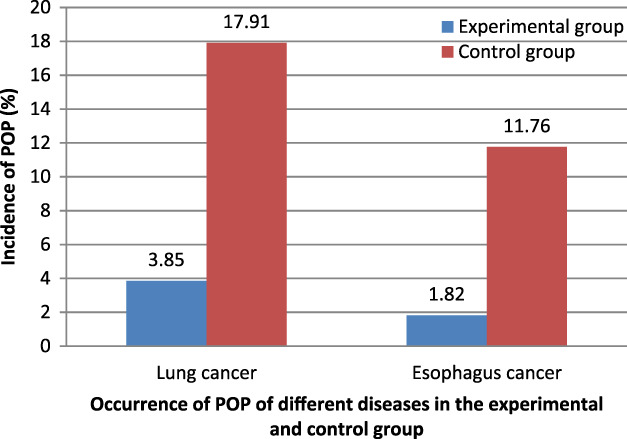
Occurrence of postoperative pneumonia (POP) of different diseases in the experimental and control groups. *Significant difference between two groups by *X* ^2^ test of Mantel–Haenszel–Cochran (*p* < 0.05)

In the experimental group, three out of 78 lung cancer patients had POP, while in the control group, 12 out of 67 patients had POP. Among patients with esophageal cancer, one case of POP occurred in 55 patients in the experimental group, and six cases of POP occurred in 51 patients in the control group. There was a statistically significant difference in POP incidence between the experimental and control groups (*p* < 0.05; Figure 2 and Table 4).

**FIGURE 3:**
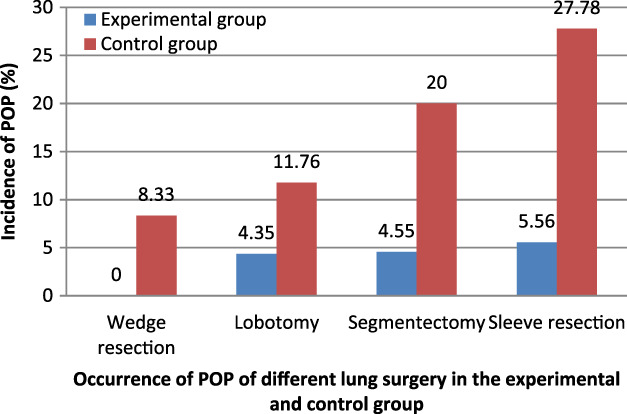
Occurrence of postoperative pneumonia (POP) of different lung surgery in the experimental and control groups. *Significant difference between two groups by *X* ^2^ test of Mantel–Haenszel–Cochran (*p* < 0.05)

In the experimental group, no patients with POP underwent wedge resection, one underwent lobectomy, one underwent pulmonary segmentectomy, and one underwent sleeve resection, respectively. In the control group, one patient with POP underwent wedge resection, two underwent lobectomy, four underwent pulmonary segmentectomy, and five underwent sleeve resection, respectively. There was a statistically significant difference in POP incidence between the two groups (*p* < 0.05; Figure 3 and Table 4).

**FIGURE 4:**
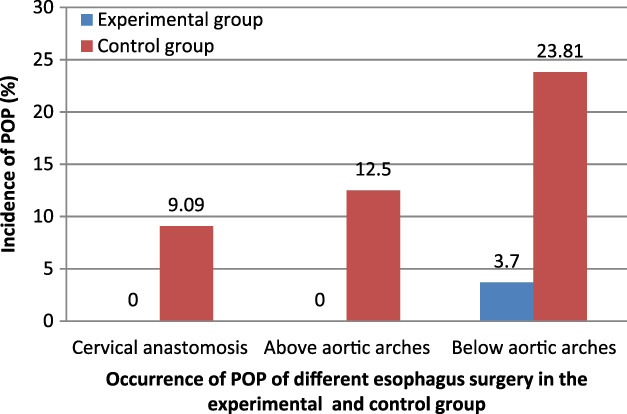
Occurrence of postoperative pneumonia (POP) of different esophageal surgery in the experimental and control groups. *Significant difference between two groups by *X* ^2^ test of Mantel–Haenszel–Cochran (*p* < 0.05)

Patients with POP had cervical anastomosis after esophageal resection (1), above the aortic arch (2), and below the aortic arch (3) in the control group. Only one patient with POP had cervical anastomosis below the aortic arch in the experimental group. There was a statistical difference in the incidence of POP between the experimental and control groups (*p* < 0.05; Figure 4 and Table 4).

## DISCUSSION

Previous studies have reported many methods to reduce the incidence of POP, such as video‐assisted thoracoscopic surgery (VATS), prophylactic antibiotics, and airway management. However, open thoracotomy is still an important surgical procedure, and its incidence has not significantly decreased.^22^ Periodontal disease is related to some respiratory diseases,^19^ but most studies have not examined its effect on POP incidence. The insufficient data collected in this study may be an important factor affecting the prevention of POP. This study discusses the effect of treating chronic periodontitis on the reduction of POP incidence.

Chronic periodontitis is initiated and sustained by dental plaque biofilm, smoking, poor oral hygiene, and dentists not discussing the formation and accumulation of dental plaque. Dental plaque adheres tightly to and between teeth and cannot be easily washed away by vigorous rinsing or water spray. It also resists disruption by antimicrobial agents that cannot easily penetrate the protective polysaccharide matrix barrier characteristic of biofilm.^19^ Pathogenic respiratory bacteria, such as lichen in the subgingival dental plaque of periodontal pockets, are seldom eliminated by applying prophylactic antibiotics following open thoracotomy. This is because it is not possible to achieve effective antirespiratory pathogenic bacterial concentrations of antibiotics in dental plaque.^17^ However, pathogenic respiratory bacteria in the periodontal pocket can be cleared following the nonsurgical treatment of chronic periodontitis. The term nonsurgical therapy includes the use of oral hygiene self‐care, periodontal instrumentation, and chemotherapeutic agents to prevent, arrest, or eliminate periodontal disease. Scaling, root planing, and polishing using periodontal instrumentation can remove the plaque and take on the form of dental calculus. Thus, along with removing dental plaque, the plaque‐harboring respiratory pathogenic microbes, cytokines, and enzymes they induce are eradicated, and the microniches regathered are destroyed. With daily oral hygiene self‐care such as toothbrushing and flossing, dental plaque can be effectively controlled. Antimicrobial peptide periodontal gel, used in the present study, is an antibacterial peptide developed according to the salivary histatin. It is not an antibiotic agent and has no side effects. If placed directly into the periodontally diseased pocket, it can repair the imbalance in the oral ecological system, neutralize the bacterial endotoxin produced by periodontal bacteria, eliminate the inflammation caused by oral treatment, and effectively kill various oral pathogenic bacteria, *Candida* and fungi. The slow‐release formulation of the product can gradually release the antibacterial peptides into the local area to constantly control pathogenic bacteria and promote the restoration of the periodontal tissue and mucosa. The application of antimicrobial peptides in treating periodontitis with different pocket depths can achieve a better curative effect.^23^

In this study, POP incidence was 3.01% in patients in the experimental group that underwent periodontal treatment. This incidence rate is similar to other studies previously reported in the literature,^24^ ^,^ and is significantly lower than that in the control group without periodontal treatment and lower than the range of 12.0% to 17.5% that has been previously reported.^22^ With periodontal therapy, the pathogenic respiratory bacteria were eliminated, and periodontally diseased pockets disappeared. The study shows that periodontal therapy was associated with a lower incidence of POP following lung and esophageal surgery, and thus, improved periodontal condition assists in the prevention of POP.

Patients after lung or esophageal cancer surgery may present with a number of related complications. As such, it can be challenging to determine whether a patient's pulmonary complications are caused by periodontitis as opposed to surgical complications. In our study, some factors may have affected the results (as mentioned in the methods section), and some participants were excluded (as described in the results section). In addition, the incidence of POP at each level was analyzed statistically in different disease or surgical methods between the experimental and control groups. We found that POP incidence in the experimental group was lower than that in the control group with a statistical difference (*p* < 0.05). Therefore, no differences were found between patients with lung or esophageal cancer, different surgical procedures, the presence of mild, moderate, or severe periodontitis in relation to POP occurrence. The presence of periodontitis is an important predisposing factor for POP in patients following open chest surgery.

However, the incidence of POP was 3.01% in the experimental group with periodontal treatment. The risk factors of POP that have been previously reported include the presence of underlying diseases such as chronic lung disease, congestive heart failure, or DM; age > 70 years, mechanical ventilation or intubation, a history of smoking, previous antibiotic treatment, immunosuppression, a long preoperative stay, and prolonged surgical procedures.^25^, ^26^, ^27^, ^28^ These factors were controlled as much as possible, and the bacteria in the air of the hospital unit was supervised and sterilized regularly. It has been previously reported that POP may be caused mainly by drug‐resistant pathogenic bacteria colonized in the airway because respiratory pathogenic microbes colonized in the airway are related to POP incidence.^29^ This suggests that periodontal condition improvement combined with airway management could help prevent POP in patients undergoing open thoracotomy. This should be explored further in the future.

Three participants in the experimental group had gingival bleeding or dental plaque at the oral visits after surgery and were subsequently considered to have periodontitis. However, they did not have POP. It is challenging to evaluate the influence of periodontitis on POP, and therefore, these patients were excluded from the study.

There are some limitations to the present study. First, our sample size was small. Thus, a large sample is required to evaluate the influence of disease species and surgical procedure on POP in each group. Second, the pathogenic bacteria in the airway cannot be controlled, which may influence the percentage of POP in our study. Third, because groups were assigned according to patient preference, the results may not have accurately reflected the actual situation of all patients with lung or esophageal cancer. Fourth, this study included only patients without common comorbidities such as DM and COPD. The effect of preoperative periodontal treatment on patients with complicated comorbidities was not explored. In addition, the effects of some factors, such as age, sex and smoking, were not further analyzed.

In conclusion, periodontal treatment was associated with a lower incidence of POP following lung or esophageal cancer surgery. Improving the periodontal condition contributes to the prevention of POP. The presence of periodontitis is an important predisposing factor for POP in patients following open chest surgery. It is recommended that periodontal examination and therapy be carried out before commencing lung or esophageal cancer surgery.
